# Cutting‐Edge Progress in Aqueous Zn‐S Batteries: Innovations in Cathodes, Electrolytes, and Mediators

**DOI:** 10.1002/smll.202405810

**Published:** 2024-10-04

**Authors:** Tianyue Liang, Xinren Zhang, Yixuan Huang, Yile Lu, Haowei Jia, Yu Yuan, Linghui Meng, Yingze Zhou, Lu Zhou, Peiyuan Guan, Tao Wan, Michael Ferry, Dewei Chu

**Affiliations:** ^1^ School of Materials Science and Engineering University of New South Wales Sydney 2052 Australia

**Keywords:** additive engineering, cathode modification, energy storage, redox mediator electrolytes, zinc‐sulfur batteries

## Abstract

Rechargeable aqueous zinc‐sulfur batteries (AZSBs) are emerging as prominent candidates for next‐generation energy storage devices owing to their affordability, non‐toxicity, environmental friendliness, non‐flammability, and use of earth‐abundant electrodes and aqueous electrolytes. However, AZSBs currently face challenges in achieving satisfied electrochemical performance due to slow kinetic reactions and limited stability. Therefore, further research and improvement efforts are crucial for advancing AZSBs technology. In this comprehensive review, it is delved into the primary mechanisms governing AZSBs, assess recent advancements in the field, and analyse pivotal modifications made to electrodes and electrolytes to enhance AZSBs performance. This includes the development of novel host materials for sulfur (S) cathodes, which are capable of supporting higher S loading capacities and the refinement of electrolyte compositions to improve ionic conductivity and stability. Moreover, the potential applications of AZSBs across various energy platforms and evaluate their market viability based on recent scholarly contributions is explored. By doing so, this review provides a visionary outlook on future research directions for AZSBs, driving continuous advancements in stable AZSBs technology and deepening the understanding of their charge–discharge dynamics. The insights presented in this review signify a significant step toward a sustainable energy future powered by renewable sources.

## Graphical abstract

Current challenges for aqueous Zn‐S batteries include volume expansion, slow kinetics, low conductivity, and side reactions, affecting their performance and stability. However, ongoing advancements hold great promise, with potential applications in areas like wearable electronics, portable devices, electric vehicles, and freeze‐resistant energy storage, pushing Zn‐S batteries toward practical use as next‐generation energy solutions.

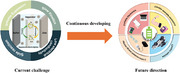

## 1 Introduction

People are feeling compelled to speed up the investigation of energy storage devices due to the escalating depletion of fossil fuel reserves and the urgent problem of environmental pollution.^[^ ^1^ ^]^ Among which, rechargeable batteries have long permeated human daily life to meet the demand for social development.^[^ ^2^ ^]^ Currently, lithium‐ion batteries (LIBs) are the predominant choice for powering electronic devices and electric vehicles in terms of energy supply applications.^[^ ^3^ ^]^ Although optimized LIBs can currently reach specific energies (E) of ≈200 Wh kg^−1^, the energy density is not yet satisfactory.^[^ ^4^ ^]^ Furthermore, safety concerns is a lingering problems due to the flammable organic electrolytes and the heat releasing when electrochemical reactions occur.^[^ ^5^ ^]^

**Figure 1:**
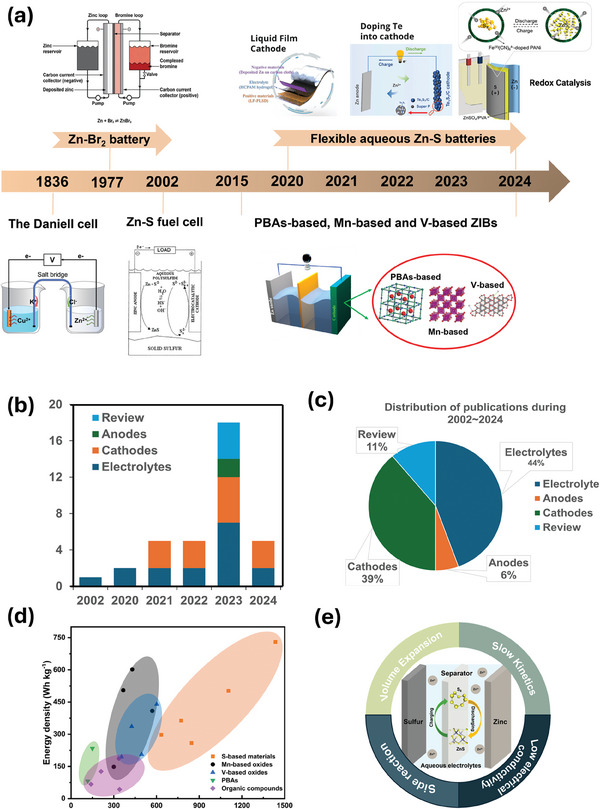
a) Main progress of Zn‐based batteries: from 1826 to now. Development of the Daniell cell, reproduced with permission from.^[^ ^13^ ^]^ Copyright 2023 RSC; development of Zn‐Br2 battery, reproduced with permission from.^[^ ^14^ ^]^ Copyright 2018 Elsevier; development of Zn‐S fuel cell, reproduced with permission from.^[^ ^15^ ^]^ Copyright 2002 ACS; development of flexible aqueous Zn‐S batteries, reproduced with permission from.^[^ ^12^, ^16^ ^]^ Copyright 2020 Wiley, 2021 ACS and 2024 Wiley, respectively; development of PBAs‐based, Mn‐based and V‐based ZIBs, reproduced with permission from.^[^ ^17^ ^]^ Copyright 2021 Elsevier, 2018 Wiley, 2022 Elsevier and 2024, IOP, respectively. b,c) Distribution of publications during 2002–2024. d) comparison of the specific capacity of different cathode materials used in zinc‐ion batteries. S‐based materials reproduced with permission from.^[^ ^2^, ^18^ ^]^ Copyright 2020 Wiley, Copyright 2023 Wiley, Copyright 2023 CAS, Copyright 2021 ACS, Copyright 2022 Elsevier, Copyright 2023 NPJ, respectively; Mn‐based materials reproduced with permission from:.^[^ ^19^ ^]^ Copyright 2017 Wiley, Copyright 2019 Wiley, Copyright 2022 Wiley, Copyright 2021 Wiley, respectively; V‐based materials reproduced with permission from,^[^ ^2^, ^20^ ^]^ Copyright 2019 Wiley, Copyright 2019 AAAS, Copyright 2021 ACS, Copyright 2019 Wiley, respectively; PBAs reproduced with permission from,^[^ ^21^ ^]^ Copyright 2022 RSC, Copyright 2019 Wiley, respectively; Organic compounds reproduced with permission from,^[^ ^18^, ^22^ ^]^ Copyright 2023 Wiley, Copyright 2022 Wiley, Copyright 2020 Wiley, Copyright 2022 Wiley, respectively. e)The schematic diagram of mechanism illustrates the aqueous Zn–S batteries.

To enhance the electrochemical performance of LIBs, lithium‐sulfur batteries (LSBs) are viewed as a promising future battery technology because of the substantial theoretical battery capacity of 1667 mAh g^−1^ and the theoretical energy density reaching up to 2600 Wh kg^−1^.^[^ ^6^ ^]^ Unfortunately, LSB systems are beset by several significant challenges including the flammability and toxicity issues associated with organic electrolytes and the increasing rarity of lithium resources, these factors contribute to elevated costs, acting as obstacles to its practical implementation.^[^ ^7^ ^]^ Hence, aqueous zinc‐ion batteries (AZIBs) have garnered considerable interest in addressing safety concerns (**Figure** **1**a).^[^ ^8^ ^]^ In the case of the battery being short‐circuited, safety concerns such as fire or explosion do not arise due to the electrolyte being aqueous.^[^ ^9^ ^]^ The numerous advantages include abundant availability (≈300 times greater than lithium), low redox potential (−0.76 V versus SHE), higher ionic conductivity (≈6 S cm^−1^), and substantial theoretical capacity of 820 mAh g^−1^.^[^ ^10^ ^]^ The cost of AZIBs anodes (≈$ 2.4 kg^−1^) are much lower than LIBs (≈$ 19.2 kg^−1^).^[^ ^11^ ^]^

Regrettably, either LSBs or AZIBs hardly offers promoted energy density and high safety simultaneously.^[^ ^8a,b^ ^]^ Consequently, a growing interest in high‐capacity, non‐toxic, and cost‐effective aqueous batteries exhibit particularly appealing.^[^ ^8a,b^ ^]^ Recent advances in studying aqueous zinc‐sulfur batteries (AZSBs) have positioned them as a frontrunner for various energy storage applications (Figure 1b,c). Highlighted by their cost‐effectiveness, enhanced safety features, larger capacity relative to other materials (Figure 1d), AZSBs demonstrate significant potential in energy storage.^[^ ^4^, ^12^ ^]^ The working mechanism of AZSBs combines the features of both AZIB and LSBs, i.e., entailing the movement of Zn^2+^ within the aqueous electrolyte, and the conversion reactions taking place on the sulfur (S) cathode.^[^ ^2h^ ^]^

Admittedly, the benefits of using AZSBs are indeed greater than other batteries, especially in terms of energy density (**Table** **1**), but AZSBs have encountered considerable obstacles that have hindered their progress toward further commercial applications. At present, each component of AZSBs mainly face the following challenges:

**Table 1:** Comparison of the electrochemical performances of different metal‐based batteries.

Battery type	Average voltage [V]	Energy density [Wh kg^−1^]	Life cycle	Self‐discharge/month [room temp]	Refs.
Lead‐acid	2	30–40	200–300	5%	[35]
Ni‐Cd	1.2	40–60	1000	20%	
Ni‐MH	1.2	60–100	300–500	30%	[36]
Li‐ion	3.3‐3.8	150–200	500–2000	<10%	[37]
Li‐S	2.1	200–400	50–100	15%	[38]
Zn‐ion	1.4	50–300	>1000	–	[39]
Zn‐S	2.4	250–800	50–300	–	[16, 18]

1) *Anode*: Dendrite formation and corrosion of Zn anode: Zn metal is susceptible to corrosion and unregulated growth of dendrites.^[^ ^23^ ^]^ The primary factors supplying dendrite growth are uneven electric field distribution and ion flux across the interface.^[^ ^24^ ^]^ These dendrites maybe detach, reduce CE, and potentially cause short circuits due to the continuous development and uneven deposition on the anode surface, compromising limited reversibility.^[^ ^24^, ^25^ ^]^

2) *Electrolyte*: Hydrogen evolution reaction (HER): Water is generally used as the solvent for AZSBs. However, its low decomposition voltage reduces the electrochemical window. This limitation hampers the utilization of cathodes high voltage and restricts the energy density of the battery to a lower threshold.^[^ ^26^ ^]^ Since the potential of hydrogen evolution (0 V vs SHE) exceeds standard electrode potentials of Zn/Zn^2+^ (−0.76 V), HER is an inevitable occurrence that reduces the Coulombic efficiency (CE) through continuous Zn metal loss during anode plating/stripping.^[^ ^27^ ^]^ The zinc anode is surrounded by precipitated hydrogen gas, which impedes the nucleation of zinc, resulting in elevated overpotentials.^[^ ^28^ ^]^ Over time, hydrogen evolution consumes the water in the electrolyte and accelerates the hydration of Zn^2+^, leading to increased zinc loss.^[^ ^29^ ^]^ The substantial production of H_2_ gas concurrently raises the internal pressure within the battery, potentially leading to the expansion and rupture of the battery.^[^ ^30^ ^]^ To decrease these risks, it is crucial to meticulously choose the suitab electrolyte and broaden the electrochemical window, which effectively decreases gas production. A detailed explanation of these strategies follows.

3) *Cathode*:

a) *Slow Kinetics*: Although the transition of the S cathode in the aqueous electrolyte may circumvent the shuttle effect due to the insolubility of ZnS in organic electrolytes, the poor wettability with the aqueous electrolyte of the cathode obstructs the transport of Zn^2+^ ions.^[^ ^31^ ^]^ This ultimately results in sluggish kinetics of the cathode electrode.

b) *Low electrical conductivity of S and its discharge byproducts*: At room temperature, sulfur exhibits extremely limited electrical conductivity (5 × 10^−28^ S m^−1^) as well as its discharging products ZnS (10^−9^ S m^−1^) are fundamentally poor.^[^ ^16^, ^32^ ^]^ The low electrical conductivity leads to huge internal resistance and polarization, resulting in sluggish reaction kinetics and a decline in the overall performance of the AZBs.^[^ ^18e^ ^]^ Simultaneously, the ZnS exists on the anode surface as a passivation film effectively inhibiting subsequent discharge.^[^ ^33^ ^]^

c) *Volume expansion at the cathode*: Sulfur experiences significant volume changes (50.3%) during cycling, which can lead to mechanical destruction to the electrode, causing a decline in cycling stability and rate performance.^[^ ^34^ ^]^

The development of AZSBs is in its nascent phase, facing numerous challenges. Thus, reviewing and discussing prior research is essential for enhancing the performance and practicality of AZSBs, aiding in strategizing solutions to overcome these obstacles, and boosting the efficiency and feasibility of battery systems. There have been excellent reviews about AZSBs, such as Patel's group and Feng's group, which have primarily concentrated on enhancing zinc anode protection and electrolyte optimization.^[^ ^40^ ^]^ In this review, we discussed the reaction mechanism and focused on classifying and analyzing the modification of cathode materials. It collects and integrates complex electrochemical properties and parameters related to testing conditions for batteries to explore their impact on the charge storage mechanisms and kinetic characteristics of AZSBs. Subsequently, all gathered information and data are comprehensively analyzed and graphically presented to illustrate the advancements in this field, emphasizing the progress of research rather than just elaborating exhaustive details for each case or solely reviewing strategies for optimizing battery performance. And proposed future research directions and prospects, which provide valuable insights into the sustainable development of AZSBs, especially concerning cathode performance enhancement.

## 2 Working Mechanism of AZSBs

Understanding the mechanism of AZSBs is essential for advancing the performance for each component. Typically, AZSBs feature a Zn anode, a sulfur‐based cathode, and an aqueous electrolyte (Figure 1e). Like AZIBs, Zn anode is employed because of its cost‐effective, high theoretical capacity, and favorable reversibility in aqueous electrolyte. During discharge, Zn will be oxidized and forming Zn^2+^ ions stripping from the anode surface, which migrate through the electrolyte.^[^ ^41^ ^]^ When charging, Zn^2+^ will be plated back onto the surface of anode after absorbing electrons. The charge–discharge formulae of Zn anode are presented below:

(1) $$\mathrm{Discharging}:\;\mathrm{Zn}-2e^{-}\to Zn^{2+}$$

(2) $$\mathrm{Charging}:\;Zn^{2+}+2e^{-}\to \mathrm{Zn}$$

In this context, Zn anodes encounter various problems extensively studied recently, including dendrite formation and anode‐related reactions such as corrosion and HER.^[^ ^42^ ^]^ Since the modification of Zn anodes has been extensively studied and reported in the study of AZIBs, and AZSBs and AZIBs share similar Zn‐based mechanisms and are therefore not discussed in detail. Wang et al. conducted a comprehensive review of the challenges associated with Zn anode design strategies for AZIBs. For an in‐depth discussion of the anode, readers are referred to their article and will not be elaborated in this article.^[^ ^23^, ^43^ ^]^

**Figure 2:**
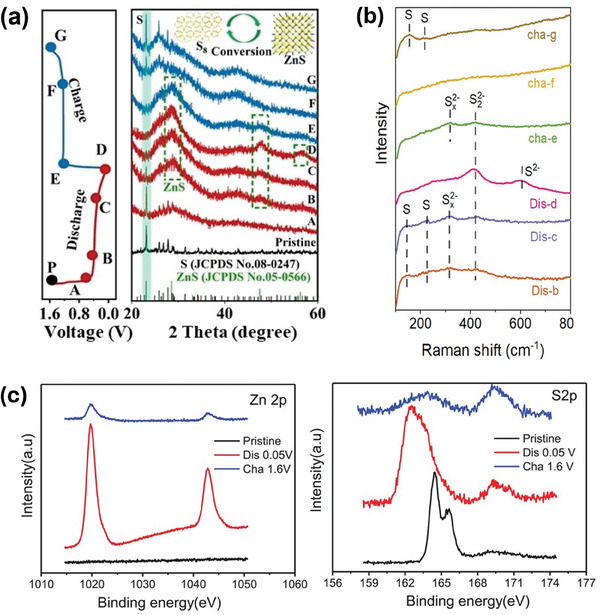
a) XRD patterns of the S cathode at different specific states during discharging/charging. The marked dots in the left diagram correspond to the states selected for the XRD tests, reproduced with permission from.^[^ ^31^ ^]^ Copyright 2022 Wiley. b) Raman spectra of S cathode at different specific states during discharging/charging, reproduced with permission from.^[^ ^45^ ^]^ Copyright 2023 Wiley. c) XPS spectra of Zn 2p and S 2p at the pristine, discharged and charged states, respectively, reproduced with permission from.^[^ ^2h^ ^]^ Copyright 2020 Wiley.

Unlike the insertion/extraction type of the Zn anode, the S cathode region undergoes a reversible conversion‐type electrochemical mechanism during cycling. Ideally, in the discharge phase, Zn^2+^ is incorporated with the S cathode in the electrolyte, facilitating the growth of ZnS accepting of Zn^2+^ ions and electrons.^[^ ^44^ ^]^ The charging procedure is the reverse of the discharging process (converting ZnS back into S and Zn^2+^). Yang et al. suggested a simple electrochemical process at the cathode that involves a direct transition between S and ZnS without forming intermediate polysulfides, which evidenced by XRD analysis and a lack of polysulfide signals in Raman spectroscopy.^[^ ^31^ ^]^ As demonstrated in **Figure** **2**a, the XRD results presented that the main peak intensity of sulfur progressively reduced during discharge. As the battery was further discharged and fully discharged, the peaks of sulfur have entirely disappeared and the peak of ZnS can be observed. As the battery charged again, the peak intensity of ZnS gradually diminished, and the intensity of sulfur became stronger. The electrochemical redox of S is listed below:

(3) $$\mathrm{Discharging}:\;S+Zn^{2+}+2e^{-}\to \mathrm{ZnS}$$

(4) $$\mathrm{Charging}:\;\mathrm{ZnS}-2e^{-}\to S+Zn^{2+}$$

However, the work of Guo et al. supported by Raman analyses (Figure 2b), indicates the formation of polysulfides of varying chain lengths during the S to ZnS conversion.^[^ ^45^ ^]^ During the cathode discharge process, three different peaks appeared at 317, 420, and 610 cm^−1^, which may indicate S_x_^2−^, S_2_^2−^, and sulfide S^2−^, respectively. A part of the S_2_^2−^ further transitions to S^2−^ (in state d). Then, the S_x_^2−^peak reemerged in the charging state (d → g). Upon further charging to state g, both S_2_^2−^ and S_x_^2−^ peaks vanish, and the two peaks of elemental S_8_ reappear, indicating a multi‐phase reaction process (S_8_→ S_x_^2^ → S_2_^2−^ + S^2−^). This aligns with the findings of Gross et al., which describe a discharge curve with two distinct plateaus indicative of a two‐step conversion process: initially from S^2−^ to S_4_^2−^, followed by a transition from S_4_^2−^ to elemental sulfur.^[^ ^46^ ^]^ To resolve these discrepancies and accurately delineate the cathode reaction mechanism in AZSBs, employing ex‐situ/in‐situ X‐ray Absorption Spectroscopy (XAS) is recommended. XAS is mostly used in LSBs to detect polysulfide generation and transformation.^[^ ^47^ ^]^ It may provide critical insights into the cathode reactions of AZSBs.

In addition, Li et al. revealed the XPS results of S after cycling (Figure 2c), which implied that S could also be partially converted into SO_4_^2−^ within the aqueous electrolyte.^[^ ^2h^ ^]^ This also explains the reason for the capacity decline and the reduced battery reversibility due to formation of unusable SO_4_^2−^. The comprehensive electrochemical reactions can be outlined as follows:

(5) $$2\mathrm{ZnS}+4H_{2}O-10e^{-}\to 2Zn^{2+}+S{O_{4}}^{2-}+S+8H^{+}$$

It is worth noting that the densities of sulfur (2.3 g cm^−3^) and ZnS (4.09 g cm^−3^) are significantly different, which will induce a great volumetric variation up to 50.3% during the electrochemical reactions.^[^ ^16a^ ^]^ Such volumetric change causes the electrode to undergo pulverization, forming an isolated area. This part will be described in detail in the next chapter.

## 3 Design Strategies for the S Cathode

### 3.1 Key Challenges

Elemental sulfur is the predominant cathode material employed in metal‐S batteries due to sulfur cathodes providing a cost‐effective solution with high energy density. Sulfur differs from intercalation compounds as it lacks active intercalation sites. In the electrochemical discharge process, the entire bulk of elemental sulfur can undergo a reaction.^[^ ^48^ ^]^ However, the inherent insulative properties of sulfur present difficulties in metal‐S batteries.^[^ ^40a^ ^]^ The inadequate conductivity in S and metal sulfide brings notable internal resistance hampering the charge transfer and reaction kinetics, further resulting in notable battery polarization, particularly at elevated C‐rates.^[^ ^49^ ^]^ To overcome these obstacles, S cathode usually utilizes a physical blend with binder materials and a conductive carbon‐based matrix (such as CNT, carbon black, and activated carbon), which is a benefit to enhance performance sulphur composites for cathodes (Detailed discussion in the section 3.2).^[^ ^50^ ^]^

In addition to the low conductivity issue, the volume expansion of the S cathode is also a challenge. When S is used in the Na‐S batteries cathode, the volume expands by 260% when converted to Na_2_S, and 80% when converted to Li_2_S in LSBs. Although the volume grows by only 50% when converted to ZnS in AZSBs, the process still causes S to separate from the cathode and pulverize, resulting in irreversible capacity loss.^[^ ^34^, ^51^ ^]^ The design of S‐based cathodes prioritizes superior porosity to manage volume changes.^[^ ^40a^ ^]^ In Zn‐S batteries, challenges except for poor sulfur conductivity and volume fluctuations, also involve slow kinetics of AZSBs. In aqueous electrolytes, due to the interaction of ZnS with H_2_O and the oxidation of sulfur from the cathode, irreversible products produced (sulfate ions: SO_4_^2−^) by side reactions can lead to S consumption and loss of active materials, reducing battery performance.^[^ ^2^, ^52^ ^]^ Therefore, overcoming S‐related difficulties is crucial to improving Zn‐S batteries.

### 3.2 Combining with Host Materials

As mentioned above, to improve the electronic conductivity of S, a general strategy has been verified based on several reported metal‐sulfur primary cells (Al‐S, Li‐S), which is combining S with more conductive carbon host materials. Luo et al. designed to introduce S into Ketjenblack (KB) for the creation of a sulfur cathode, thus enhancing high electronic conductivity to the cathode.^[^ ^53^ ^]^ In 1 M ZnCl_2_ aqueous solution (pH = 4.08), the primary Zn‐S batteries can provide a stable discharge plateau (0.7 V) and high capacity, which is ≈1668 mAh g^−1^. Remarkably, the sulfur cathode showcased the ability to function at elevated maximum loading mass levels of 8.3 mg as retaining a specific capacity of 1375 mAh g^−1^. Unfortunately, due to the considerable voltage lag, the circle‐trip battery efficiency is extremely low (30%). At a similar juncture, Guo et al. opted to heat a combination of nanoporous carbon (NPC) and sulfur powder to produce a cathode. Utilizing its distinctive hybrid aqueous system, the battery showed a supreme capacity of 1435 mAh g^−1^.^[^ ^45^ ^]^

Li et al. introduced sulfur‐loaded carbon nanotubes (S@CNTs) as the cathode and investigated the effect of sulfur content on electrochemical properties.^[^ ^2h^ ^]^ In S@CNTs composites, when other conditions remain unchanged, AZSBs with 50 wt% sulfur content displayed a remarkably superior capacity of 1105 mAh g^−1^ and a maximum energy density of 502 Wh kg^−1^ at 100 mA g^−1^.^[^ ^2h^ ^]^ Zhu et al. employed a template approach to encase sulfur powder within hollow carbon spheres to fabricate the cathode, revealing an elevated capacity (775 mAh g^−1^ at 2 A g^−1^).^[^ ^31^ ^]^ However, the dissatisfaction arises from the intricate process involved in producing hollow nano‐carbon spheres, along with the expensive and limited yield of carbon nanotubes (CNTs). In practical applications, the economic benefits are not satisfactory.

**Figure 3:**
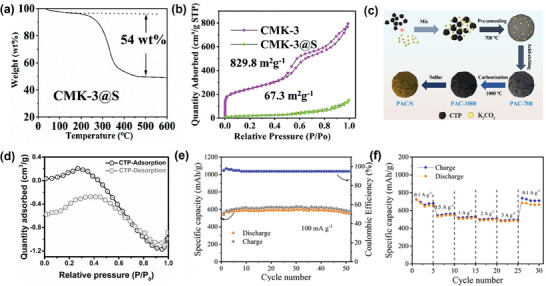
a) The thermogravimetric analysis curve for the CMK‐3@S composite. b) N_2_ adsorption‐desorption isotherms comparing CMK‐3 and the CMK‐3@S composite, reproduced with permission from.^[^ ^54^ ^]^ Copyright 2022 RSC Publishing. c) The schematic diagram of the synthesise of PAC/S. d) N_2_ adsorption‐desorption isotherm of PAC, reproduced with permission from.^[^ ^18b^ ^]^ Copyright 2023 Springer. e) Cycling performance of HMCs‐3@S at 0.1 A g^−1^. f) Rate performance of HMCs‐3@S, reproduced with permission from.^[^ ^57^ ^]^ Copyright 2023 Wiley.

Besides, Xu et al. tried to use a CMK‐3 (hexagonal ordered mesoporous carbon) as the host material for the encapsulation of sulfur as the AZSBs cathode.^[^ ^54^ ^]^ As depicted in **Figure** **3**a, the sulfur content within CMK‐3@S is determined as 54 wt% through calculation. Also, based on the N_2_ adsorption and desorption isotherms (Figure 3b), the specific surface area was decreased from 829.8 m^2^ g^−1^ (pure CMK‐3) to 67.3 m^2^ g^−1^ (CMK‐3@S), which further strongly proves that sulfur has been successfully loaded.

Apart from incorporating sulfur with CNT and CMK, Yang et al. used hollow carbon spheres (HSC) synthesized from tetraethyl orthosilicate (TEOS) and ammonium hydroxide as host materials for S loading.^[^ ^31^ ^]^ HCS/S with varying sulfur contents (HCS/S‐53.7 and HCS/S‐65.3) were obtained by cutting the glass tube. Paired with its development of a “cocktail‐optimized” aqueous solution comprising the co‐solvents (G4 and water) along with iodine (I_2_) as an additive, a great capacity of 775 mAh g^−1^ was achieved (at 2 A g^−1^) and maintaining more than 70% capacity after 600 cycles (at 4 A g^−1^). Although the application of carbon host materials such as KB, CNTs, and HSC has improved the conductivity of the cathode, the impact of its pore size on the cathode is still lacking. Hu et al. examined the effect of pore size on electrochemical performance and obtained the result that compared with mesoporous carbon, microporous carbon structure has a higher ability to confine sulfur.^[^ ^55^ ^]^

Coal tar pitches have become a focal point for researchers aiming to refine carbon host materials due to their abundant obtainability, cheap, and high carbon yield, drawing significant interest.^[^ ^56^ ^]^ As a by‐product of the coking, the development of coal tar pitch is important in efficiently utilizing waste resources. Currently, KOH and ZnCl_2_ are the primary activators to generate carbon materials by pitch as raw materials. However, these chemicals are extremely eroded and environmentally unfriendly. Therefore, Wang et al. constructed a method (Figure 3c) using K_2_CO_3_ as the activator and (CTP) as the precursor to prepare 3D amorphous carbon encapsulating S powder with porous structure as the cathode because K_2_CO_3_ is moderate, eco‐friendly, and cost‐effective.^[^ ^18b^ ^]^ This innovative approach yields a poly AlCl_3_ (PAC)/S‐60.33% cathode that demonstrates impressive electrochemical performance, including a great capacity of 633.5 mAh g^−1^ (at 0.5 A g^−1^), substantial energy density of 297.5 Wh kg ^−1^, and decent cycling stability after 400 cycles (180 mAh g^−1^ at 5.0 A g^−1^). Furthermore, evaluating surface area and pore characteristics of CTP and PAC samples using nitrogen adsorption‐desorption isotherms. BET (Brunauer‐Emmett‐Teller) results (Figure 3d) show that CTP exhibits a specific surface area of ≈0 m^2^ g^−1^, indicating a lack of pore structure. In contrast, PAC has a significant specific surface area of 682.3 m^2^ g^−1^ with an average pore diameter predominantly ≈1.7 nm, and the PAC curve exhibits a typical Type I isotherm, confirming the prevalence of a microporous structure. This process is facilitated by K_2_CO_3_, which effectively encourages the development of microporous structures within PAC.

While sulfur carriers with porous carbon as the main frameworks can largely solve the problem of poor sulfur conductivity, raw materials such as fullerene, carbon fiber, nanowires, CNTs, and graphene come from unrenewable fossil fuels, limiting development and causing environmental concerns.^[^ ^57^ ^]^ Recently, biomass‐derived carbon has attracted people's interest. Researchers have found that carbon materials derived from natural biomass can develop internal structures and undergo in‐situ self‐doping in the prepared procedure.^[^ ^58^ ^]^ The introduction of heteroatoms through doping can provide more active sites of porous materials.^[^ ^59^ ^]^ Liu et al. discovered that the N‐doped carbon material synthesized by using the natural biomass Enteromorpha as a precursor and prepared through high‐temperature carbonization and alkali treatment activation methods have the advantages of a significant specific surface area (SSA), plentiful microporous structure, and functional groups with heteroatomic elements.^[^ ^57^ ^]^ The capacity for storing charges of the prepared sulfur cathode (HMCs‐3@S) is increased, and the effective transmission of ions and electrons is also ensured. The HMCs‐3@S cathode (Figure 3e,f) exhibits considerable reversible capacity (591 mAh g^−1^ at 0.1 A g^−1^) for storing charges, excellent rate performance (477 mAh g^−1^ at 3 A g^−1^) and good cycle performance (559 mAh g^−1^ after 100 cycles at 1 A g^−1^).

Unfortunately, optimizing the conductivity of AZSBs by using carbon materials as hosts often falls short due to insufficient active sites and weak interactions.^[^ ^60^ ^]^ It can notably enhance the structural attributes of carbon materials to introduce heteroatoms. Since carbon and nitrogen have similar atomic radii, but nitrogen exhibits higher electronegativity, developing nitrogen‐doped carbon materials is a promising strategy for the conductivity problem of sulfur cathodes. The team of Xu et al. designed the carbon framework for a Zn‐S battery using density functional theory (DFT) simulation.^[^ ^61^ ^]^ Their DFT results indicated a significant increase in adsorption energy after nitrogen doping (from −0.05 to −3.60 eV), compared to the undoped carbon materials. Additionally, the potential barrier experienced a marked reduction. Nitrogen doping (NC/S) led to a continuous charge density distribution between the cathode and the polysulfides, establishing an effective electron channel for improved charge transfer. The NC/S cathode demonstrated superior electrochemical performance in tests, achieving 316.4 mAh g^−1^ at 0.2 A g^−1^.

In summary, the inherently low electrical conductivity of sulfur has been one of the challenges to overcome. Various strategies have been explored, mainly around the combination of S with conductive carbon host materials, which have shown significant improvements in the electronic conductivity of the cathode.

### 3.3 Composite Construction

While combining carbon matrices with sulfur can enhance sulfur and sulfide conductivity and prevent volume expansion, this method does not address its slow redox reactions. To tackle the issue of incomplete of sulfur conversion and the high decomposition energy barrier of the sulfur cathode during charging and discharging, researchers have explored incorporating catalysts into the cathode to modify the reaction dynamics of sulfur.

**Figure 4:**
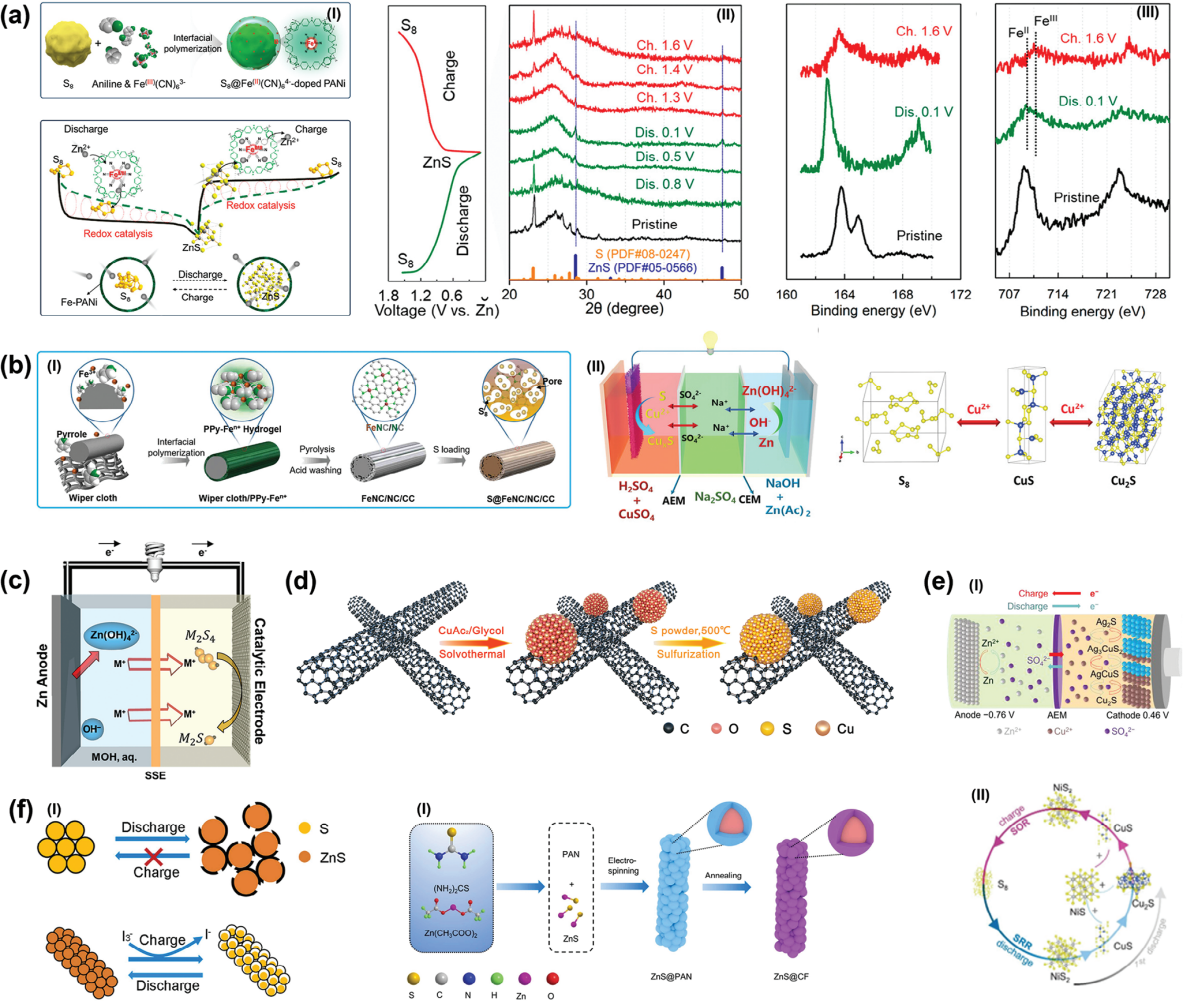
a) I) The schematic diagram of the method for fabricating the sulfur cathode involved encapsulating the sulfur nanoparticle with a flexible Fe(CN)_6_^4−^ doped polyaniline (Fe‐PANi) film; II) The schematic for sulfur redox in Zn–S cells with redox catalysis promoted an effective pathway for cation transport; III) enables the reversible conversion of sulfur, and XPS spectra of S 2p and Fe 2p of sulfur cathode with different discharge/charge states, reproduced with permission from.^[^ ^16a^ ^]^ Copyright 2022, American Chemical Society. b(I)) The schematic diagram of the synthesis procedure for S@FeNC/NC/CC through polymer coating, carbonization, etching iron species off and sulfur loading; II) The schematic diagram of the intended hybrid alkali/acid Zn–S battery and the conversion pathway of S electrode in as‐proposed h‐ZnSB, reproduced with permission from.^[^ ^62^ ^]^ Copyright 2023 Wiley. c) The schemes of the aqueous zinc‐polysulfide battery, where the alkali metal M represents is either Na or Li, reproduced with permission from.^[^ ^46^ ^]^ Copyright 2018, American Chemical Society. d) The schematic diagram of the synthesis CuSx/CNTs, reproduced with permission from.^[^ ^64^ ^]^ Copyright 2021 Elsevier. e(I)) The schematic diagram of phrase conversion in charge and discharge process; II) Schematic for phrase conversion during the cycling process, reproduced with permission from.^[^ ^66^ ^]^ Copyright 2023, Oxford University Press. f(I)) The illustration of the structure change of S and ZnS@CF cathode during the cycling process; II) the schematic illustration of the preparation of ZnS@CF, reproduced with permission from.^[^ ^18d^ ^]^ Copyright 2022, Elsevier.

In this context, Lu et al. designed an extraordinary‐capacity cathode (**Figure** **4**a) via an in‐situ interfacial polymerization to enhance the conversion kinetics of AZSBs, and studied its degradation mechanism.^[^ ^16a^ ^]^ This cathode incorporated a Fe‐based redox pair, the Fe^II^(CN)_6_^4–^/Fe^III^(CN)_6_^3–^ redox couple from open Fe species activates sulfur, reduces energy barrier for the activation of ZnS during reverse charging, facilitating 3D Zn^2+^ ion migration from Zn_x_Fe^II^(CN)_6_ to S and resulting in expediting the redox kinetics, raising capacity, and lengthening service life.^[^ ^16a^ ^]^ This achievement led to the realization of exceptional‐performance S redox chemistry directed by redox catalysis.^[^ ^34^ ^]^ Subsequent work demonstrated the AZSBs with a yolk‐shell structure containing 70 wt% sulfur S@Fe‐PANi cathode and PVA‐ZnSO_4_ gel electrolyte, using Zn_x_Fe^II/III^(CN)_6_ polymer shell serves as a cation pool, accelerating the slow redox kinetics of sulfur, increasing the reversible capacity of the battery cathode, and making the performance more stable.^[^ ^16a^ ^]^ Characterization (XRD and XPS) results show that during the reaction process, sulfur redox kinetics affect the reversibility process of S conversion to ZnS, while the Fe‐based redox couple accelerates sulfur redox kinetics.

Meanwhile, Zhang et al. used a different method to aid in the reversible conversion of sulfur throughout the cycling process.^[^ ^62^ ^]^ The Fe sites with Fe‐N_4_ coordination was utilized as bidirectional electrocatalyst sites (Figure 4b(I)), the robust interaction between the substrate and the sulfur species ensures the almost achieve transfer of S_8_ into ZnS in discharging process. Through the charging process, the activation energy barrier of zinc sulfide is lower due to the active Fe hotspots, thereby achieving high reversibility of converting ZnS into S. Despite the inadequate cycle stability of the electrode and a capacity retention rate below 60% with 300 cycles, the freestanding Fe‐embedded carbon cloth supporting the cathode still displays an extreme discharge capacity of 1143 mAh g^−1^. In summary, both methods reveal that the degradation mechanism of AZSBs is triggered by the accumulation of inactive ZnS nanocrystals. Cai et al. have also enhanced the performance of AZSBs by introducing nitrogen doping (Figure 4b(II)).^[^ ^63^ ^]^ This was achieved through the development of dispersed N‐doped hollow porous carbon (Zn‐NHPC). The presence of Zn‐N_4_ atoms on the carbon structure acts as the sulfur host. The innovative approach successfully reduces the prohibitively high barrier to vulcanization reactions that are usually encountered in N‐doped graphene (Figure 4b(IV)), leading to improved battery performance. The transformation of S and Cu_2_S takes place in the synthesized cathode immersing a CuSO_4_ solution, engaging in an electrode reaction involving four electrons that yields a substantial theoretical capacity with 3350 mAh g^−1^. Moreover, the presence of excellent electrocatalytic activity enhances the conversion efficiency of S and Cu_2_S. The Zn‐NHPC battery system proved an impressive capacity of 2250 mAh g^−1^ (at 1 A g^−1^) by matching separators, electrolytes, and an alkaline zinc anode.

Gross et al. explained the aqueous Zn polysulfide battery consisting of a catalytically active cobalt sulfide (CoS) additive cathode (Figure 4c), an aqueous polysulfide catholyte, an alkaline anolyte, a zinc anode, and a solid‐state electrolyte carrying mediator ions (Na^+^ or Li^+^).^[^ ^46^ ^]^ CoS electrocatalyst is used as a polysulfide reduction catalyst to boost cathode efficiency. The synthesis of CoS was electrodeposited cobalt on stainless steel and brass mesh substrates to form Co@SS which was then subjected to pretreatment in a 1 M Na_2_S_4_ solution to generate CoS@SS. The solid‐state electrolyte (SSE) functions as a protective barrier, efficiently segregating the reactive aqueous polysulfide catholyte from the metallic zinc anode using alkali metal ions (Na^+^ or Li^+^). This SSE prevents the polysulfide species crossover and ensures charge balance with a reversible discharge capacity of 822 mAh g^−1^ after 50 cycles with a CE of almost 100%. Besides, the findings indicate that a rechargeable zinc aqueous polysulfide (ZAPS) batteries employing Na^+^ as mediator ions exhibit superior rate and power metrics compared to Li^+^ as mediator ions. Overall, this comprehensive strategy integrating a finely crafted electrode structure with a meticulously selected electrolyte supplement, resulted in notable enhancements in reaction speed and durability over extended periods.

Beyond the realm of Co‐based, Cu‐based catalysts, Ag_2_S, and NiS_2_ catalysts also achieve the purpose of accelerating the conversion of sulfur. Qin et al. synthesized Cu_7_S_4_/CNT composites to transformation of polysulfides and accelerate S_4_^2−^ /S_2_^2−^ redox reaction (Figure 4d).^[^ ^64^ ^]^ Based on the findings of Faber et al., each film of FeS_2_, NiS_2_, and P_y_S_2_ can be identified as effective catalysts for the reduction of polysulfides, with the catalytic performance of NiS_2_ surpassing that of CoS_2_ concerning inherent activity.^[^ ^65^ ^]^ Furthermore, when the catalytic is integrated with Cu^2+^/Cu^+^, an enhancement in the energy density of aqueous batteries is attainable. For instance, Zhao et al. promoted the sulfur oxidation reaction (SOR) of mesocrystalline NiS_2_ (M‐NiS_2_) via a reversible 6‐electron redox electrochemistry (Figure 4e(I)).^[^ ^66^ ^]^ The results showed that the viable solid‐state pathway for sulfur involves S ⟷ NiS_2_ ⟷ NiS + Cu_2_S and M‐NiS_2_, effectively preventing the release of gas byproducts (H_2_S and O_2_) and side reactions. Due to the effective catalytic role of M‐NiS_2_ on sulfur, the aqueous M‐NiS_2_||Zn hybrid battery achieves the energy density to 432.9 Wh kg^−1^ and maintains 89.6% of its capacity after 250 charging cycles.

In a similar vein, Liu et al. utilized the Ag_2_S as the cathode due to excellent electronic conductivity, and Cu^2+^ as the carrier (Figure 4e(II)), to achieve a 4‐electron electrode via solid‐state translation sequence of Ag_2_S → Ag_3_CuS_2_ → AgCuS → Cu_2_S.^[^ ^67^ ^]^ This process enabled the battery to achieve a specific capacity of 432 mAh g^−1^. It still exhibited commendable cycle stability after 800 cycles. However, the higher cost of Ag_2_S is a major obstacle to its promotion. In contrast to sulfur cathodes, ZnS undergoes complete zincation and initially contracts during dezincing, creating a vacancy for the following expansion of volume during zincification, thereby alleviating structural damage.^[^ ^18d^ ^]^ Therefore, Liu et al. proposed and produced a cathode (Figure 4f) involving nanostructured ZnS particles enveloped and interconnected by a carbon sheath (ZnS@CF).^[^ ^18d^ ^]^ An efficient network of nanoscale ZnS particles synergistically combined with micron‐scale conditions ensures high capacity and substantial structural stability. ZnS@CF kept up 88% of its initial capacities after 100 cycles by an average CE of 99.87%. This is attributed to the confinement of ZnS nanoparticles within the carbon sheath to control volumetric growth.

**Figure 5:**
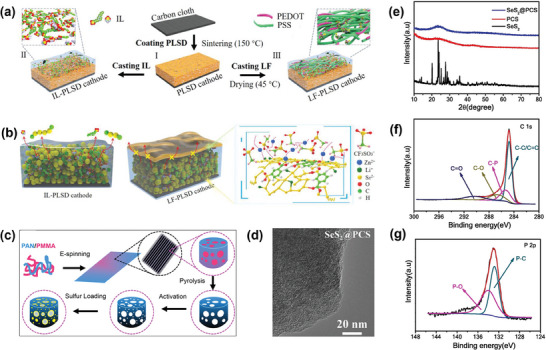
a) Schematics of synthesis process of IL‐PLSD and LF‐PLSD cathodes; b) Schematic illustration of PLSD cathodes layered with Zn^2+^conducting IL or LF. The red dashed lines signify the channels through which Zn^2+^ ions are transported in the far‐right image, reproduced with permission from.^[^ ^12b^ ^]^ Copyright 2020 Wiley. c) Manufacturing procedure for the independent CNF‐S cathode, reproduced with permission from.^[^ ^68^ ^]^ Copyright 2020 American Chemical Society. d) SEM image of SeS_2_@PCS; e) The XRD patterns of SeS_2_, PCS and SeS_2_ @PCS. f) XPS spectra of C1s and g) P2p, reproduced with permission from.^[^ ^69^ ^]^ Copyright 2022, Elsevier.

Even though redox mediators can promote reaction kinetics, further research is required to regulate the expansion of sulfur cathodes during zincification to minimize mechanical damage to the cathode and mitigate the deterioration of cycle performance. Zhao et al. detailed a dependable aqueous zinc system triggered by a “liquid film” (**Figure** **5**a) composed of 4‐(3‐butyl‐1‐imidazoline)‐1‐butanesulfonic acid ionic liquid (IL) contained in PEDOT:PSS.^[^ ^12b^ ^]^ which activates the AZSBs during cell operation. The cathode, replacing conventional solid sulfur, incorporates a poly(Li_2_S_6_‐random‐(1,3‐diisopropenylbenzene)) (PLSD) copolymer. Additionally, the electrolyte comprises 1 M zinc bis(trifluoromethylsulfonyl)imide (Zn(TFSI)_2_), and the introduction of 1,3‐diisopropenylbenzene (DIB) through robust covalent interactions serves as a polysulfide “anchor” to impede polysulfide shuttling. The need of extra binders is eliminated because the substrate (carbon cloth) forms a chemical bond with the DIB to secure the whole composite attachment. the resulted cathode with binder‐free property allowing for a high PLSD mass loading. Moreover, the CF_3_SO^3−^ anions contained in IL not only act as Zn^2+^ transfer channels but also block larger TFSI^−^ anions in the electrolyte. The liquid film (LF) contains IL sealed in the PEDOT:PSS 3D scaffold, imparting the electrode superior conductivity and exceptional electrolyte permeability. The introduction of the PEDOT:PSS skeleton that forms a three‐dimensions network fixes IL in the form of LF on the surface of the PLSD cathode, avoiding the IL coating from diffusing into the electrolyte with the entry and exit of Zn^2+^ (Figure 5b), significantly enhancing the stability of the channel effect. During charging, S_6_^2−^ is reduced to S^2−^ by Zn, and oxidized during the charging to procedure long chain ZnxLiyS_3–6_. Under dual effects of the electrolyte with high concentration decreases the solubility of polysulfides (ZnxLiyS_3‐6_) and safeguarding supplied with the LF coating, and the Zn/LF‐PLSD battery demonstrated an outstanding capacity of 1148 mAh g^−1^ at 0.3 A g^−1^ and the excellent capacity and extremely convincing outstanding stability (prolonged life of 1600 cycles).

Amiri et al. presented a method to deposit sulfur on carbon nanofibers (CNF) using an electrostatic spraying technique to achieve a broad sulfur loading of more than 60 wt%, thereby synthesizing structurally robust CNF‐S as a cathode (Figure 5c).^[^ ^68^ ^]^ A Zn‐S battery featuring an electrolyte (containing zinc acetate, ethylene glycol, and I_2_ additives) along with a CNF‐S cathode addresses the issue of poor electrochemical and mechanical performance in the battery. The stripping/plating processes of Zn^2+^ have a negligible influence on CNF. The CNF‐S electrode will not show fatigue failure after undergoing mechanical and electrochemical cycles. The assembled ZnS battery carried an elevated energy density of 283 Wh kg^−1^ and successfully powered a 1.5 V electronic watch working up to 5 h (with repeated loading conditions from 50–100 MPa).

Li et al. discovered that encapsulating SeS_2_ with phosphorus‐doped carbon sheets (PCS) to form SeS_2_@PCS enhances its performance beyond that of separately used Se and S when serving as a conversion cathode in aqueous Zn‐SeS_2_ batteries.^[^ ^69^ ^]^ The electrolyte containing 1 M ZnSO_4_ 0.1 wt% I_2_ (additive), SeS_2_@PCS achieves a remarkable capacity of 1107 mAh g^−1^, an excellent platform of 0.74 V for discharge, and low overpotential of 0.41 V. Furthermore, it presents higher energy density of 772 Wh kg^−1^. In addition, I_2_ acting as a redox mediator enhances the capacity and kinetics, SeS_2_@PCS exhibited better complete performance. TEM images expose extremely amorphous structures and abundant nanopores of PCSs (Figure 5d), demonstrating SeS_2_ nanoparticles consistently dispersed within the porous carbon sheets. The results of XRD and XPS (Figure 5e–g) also prove that during the discharge and charge process, reverse conversion between SeS_2_ and ZnSe (ZnS). These findings affirm the synergy of selenium and sulfur in the batteries. The sulfur‐selenium solid solution composite, comprising SeS_14_ @ 3D‐NPCF, SeS_5.76_ @ 3D‐NPCF, and SeS_2.46_ @ 3D‐NPCF, further advanced by Li's research group, underscores the beneficial synergy between sulfur and selenium in improving battery electrochemical performance.^[^ ^70^ ^]^ These findings reaffirm the viability of incorporating Se_x_S_y_ into the cathode of AZSBs.

In summary, the advancement of AZSBs faces several significant challenges. Principal among these is the inadequate conductivity, sluggish reaction kinetics, and significant volumetric expansion experienced by the sulfur cathode during operation, which collectively degrades battery performance. The optimized design of sulfur cathodes includes designing carbon host materials (porous carbon, carbon fiber, etc.) and catalyst materials (CoS, NiS_2_, etc.) to improve cathode conductivity and facilitate faster sulfur redox reactions. However, to achieve practical application milestones, innovative approaches must be identified and implemented to address persistent challenges related to battery stability, capacity, and energy density optimization.

## 4 Designing Approaches for Electrolytes

In the realm of AZSBs, electrolytes are indispensable in serving as the primary bridge for ion migration between the electrodes, promoting the ions migration between electrodes, influencing zinc plating/stripping reversibility, and determining the electrochemical stable potential window (ESPW).^[^ ^10^, ^71^ ^]^ Compared with organic compound electrolytes, aqueous electrolytes offer several benefits including reduced flammability, higher ionic conductivity, cost‐effectiveness, and developed safety.^[^ ^9^, ^72^ ^]^ However, their drawbacks cannot be overlooked, including a restricted electrochemical window (1.23 V), accelerated capacity fading, electrolyte decomposition (via hydrogen and oxygen evolution reactions), unsatisfactory CE, problematic side reactions on zinc anodes (like dendrite formation), cathode S is lost in the form of SO_4_^2−^, and suboptimal water wettability of the cathode.^[^ ^2^, ^30^, ^73^ ^]^ For these reasons, refining the electrochemical performance of AZSBs is pivotal, and optimization and modification of aqueous electrolytes have aroused extensive discussion and research.

Therefore, an optimal electrolyte for battery applications must fulfill several criteria to enhance performance and ensure safety. It should exhibit the maximum ionic transference number which is crucial for efficient energy transfer within the battery.^[^ ^40b^ ^]^ Additionally, the physical properties of the electrolyte, including a low melting point and a high boiling point, contribute to the battery's operational stability under varying temperature conditions. Moreover, maintaining a stable and broad electrochemical window is essential for the electrolyte to withstand high‐voltage applications and enhance the battery's overall durability and efficiency. Modification of the electrolyte encompasses several strategies: elevating its concentration, incorporating beneficial additives, using mixed electrolytes, shifting from bi‐electronic to quad‐electronic reactions, and segregating the carriers in the anolyte and catholyte.^[^ ^34^, ^73^, ^74^ ^]^ The modified aqueous electrolyte can change the reaction potential, kinetics, electrochemical window, and stability over numerous cycles.

### 4.1 Direct Modifications of Conventional Electrolytes

**Figure 6:**
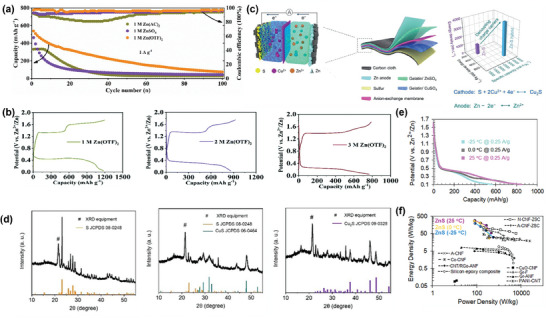
a) GCD curves of different electrolytes; b) Long‐term cycling performance of CMK‐3@NS under different concentrated electrolytes, reproduced with permission from.^[^ ^54^ ^]^ Copyright 2020 Royal Society of Chemistry. c) The illustration of the preparation and the working mechanism; d) The XRD patterns of S, CuS and Cu_2_S, reproduced with permission from.^[^ ^76^ ^]^ Copyright 2021 Wiley. e) The performance of ZnS battery during charge and discharge at different temperatures; f) The curves of energy density versus areal power density for the ZnS battery, reproduced with permission from.^[^ ^68^ ^]^ Copyright 2022, American Chemical Society.

The performance of AZSBs is determined by the complex interplay of various ions involved in their electrochemical reactions, which is significantly affected by factors such as the concentration of the electrolyte. Xu et al. explored how different electrolytes and their concentrations affect the electrochemical behaviors of AZSBs, using CMK‐3 as the sulfur‐containing host material for the cathode.^[^ ^54^ ^]^ Their results presented that AZSBs employing Zn(OTF)_2_ as the electrolyte demonstrated a better capacity retention than those of using Zn(ac)_2_ and ZnSO_4_ solution‐based electrolyte, which highlighted the significant impact of electrolyte choice on AZSB's performance. In comparing Zn(OTF)_2_ with Zn(ac)_2_ and ZnSO_4_ electrolytes (**Figure** **6**a), the presence of larger CF_3_SO^3−^ anions in the Zn(OTF)_2_ electrolyte plays a pivotal role in enhancing electrochemical performance. It is achieved by increased incorporation of H_2_O molecules within its structure. The large CF3SO3^−^ anions reduce the solvation effect of Zn^2+^, leading to better electrochemical results with Zn(OTF)_2_ electrolyte. Experimental data showed that increasing Zn(OTF)_2_ concentration positively affects the nucleation overpotential for Zn and expands the ESW, thereby minimizing HER occurrences. Concurrently, electrolyte with a high concentration diminishes corrosion on the Zn anode and augments cycling stability of the battery. Notably, at a 3 M concentration of Zn(OTF)_2_ (Figure 6b), the battery achieved an impressive capacity of 788 mAh g^−1^ and maintaining 61% capacity retention, highlighting the vital part of electrolyte concentration in enhancing battery efficiency and durability.

Another critical challenge of AZSBs lies in the conventional use of identical charge carriers within both the catholyte and anolyte, which can compromise the simultaneous optimization of energy storage performance at the electrodes.^[^ ^75^ ^]^ Dai et al. proposed a new approach for enhancing the energy storing performance of flexible aqueous batteries by strategically separating the charge carriers between the anode and cathode electrolytes.^[^ ^76^ ^]^ This method allows the Zn anode and S cathode to achieve their ideal redox reactions independently, significantly boosting the battery's energy storage capabilities (Figure 6c). Specifically, the system employed Zn^2+^ ions in the anolyte and Cu^2+^ ions in the catholyte, leveraging the inherent advantages of both the zinc anode and sulfur cathode. The reaction mechanism at the sulfur cathode is depicted through XRD analysis (Figure 6d), revealing the process as S → CuS → Cu_2_S. Additionally, SO_4_^2−^ ions facilitate charge exchange by shuttling between the anolyte and catholyte. Incorporating a flexible anion exchange membrane successfully inhibits the direct reaction between Cu^2+^ ions and the zinc anode and limits the intermixing of cations across the quasi‐solid gelatin/ZnSO_4_ anolyte and gelatin/CuSO_4_ catholyte. This setup boosts the safety features of the battery and contributes to the exceptional performance outcomes, delivering an outstanding initial discharge capacity of 3084 mAh g^−1^ S and a consistent reversible specific capacity of 2063 mAh g^−1^ S (at 100 mA g^−1^).

### 4.2 Quasi‐Solid‐State Electrolytes

As the demand for wearable electronics grows, so does the push for the advance of flexible devices. AZSBs hailed for their economical and environmentally friendly attributes, are emerging as a superior choice over the flammable and expensive lithium batteries.^[^ ^12b^ ^]^ However, the widespread adoption of AZSBs has been hampered by a critical limitation: the aqueous electrolytes freeze at subzero temperatures.^[^ ^77^ ^]^ To address this challenge, Amiri et al. have innovatively incorporated ethylene glycol (EG) into the battery design, functioning as an antifreeze agent.^[^ ^68^ ^]^ This novel hydrogel electrolyte composition includes Zn(Ac)_2_, EG, and I_2_ additives, ensuring operability and good performance even under cold conditions (Figure 6e). By utilizing a porous carbon nanofiber sulfur (CNF‐S) cathode, the quasi‐solid ZnS battery showcases impressive energy densities: achieving a maximum of 283 Wh kg^−1^ at ambient temperatures, 263 Wh kg^−1^ at 0 °C, and 193 Wh kg^−1^ at −25 °C (Figure 6f). This advancement represents a significant step forward for the applicability of AZSBs in cold environments, paving the way for more reliable sources for wearable electronic devices.

### 4.3 The Additive of Electrolytes

**Figure 7:**
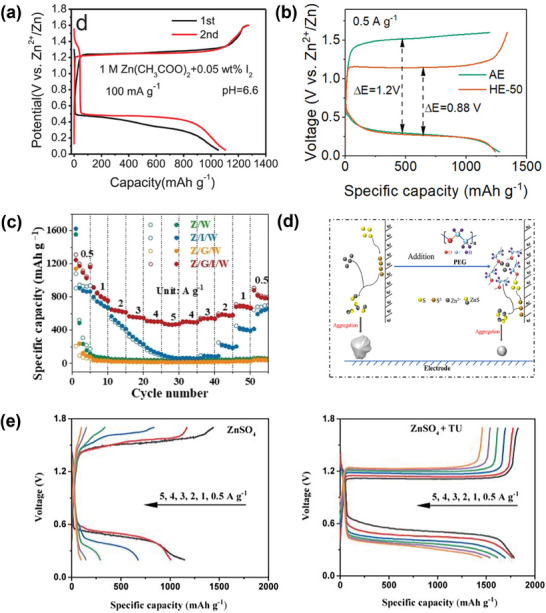
a) Charge and discharge curves of S@CNTs‐50 cathode in 1 M Zn(Ac)_2_ with 0.05 wt% I_2_ as additive, reproduced with permission from.^[^ ^34^ ^]^ Copyright 2020 Wiley b) The initial cycle of charge and discharge performance of Zn/50‐S@NPC at 0.5 A g^−1^ with various electrolytes, reproduced with permission from.^[^ ^45^ ^]^ Copyright 2023 Wiley. c) Cycling performance of the Zn‐S batteries in Z/G/I/W with a current density at 4 A g^−1^, reproduced with permission from.^[^ ^31^ ^]^ Copyright 2022 Wiley. d) Illustration of the impact of introducing PEG, reproduced with permission from.^[^ ^78^ ^]^ Copyright 2022 Elsevier. e) GCD curves of S@KB electrode under different current densities without/with TU in the electrolytes, reproduced with permission from.^[^ ^79^ ^]^ Copyright 2023 Elsevier.

1) *Iodine*: The electrochemical efficiency of AZSBs can also be significantly improved through the incorporation of additives. Incorporating redox mediators, like I_2_, into the electrolyte has emerged as a successful strategy to improve the properties of AZSBs. The interaction between I_2_/I^−^ introduces a synergy where sulfur takes on mixed properties of both I_2_ or I_3_^−^ and Sulfur. The combination facilitates the capture of Zn^2+^ ions at elevated potentials, leading to an increase in discharge potential. Adding I_2_ can expedite the oxidation process of ZnS and minimize voltage hysteresis.^[^ ^34^ ^]^ Li et al. proved the efficacy of this approach with AZSBs featuring an S@CNTs‐50 cathode, 1 M Zn(Ac)_2_ electrolyte (pH = 6.5), and 0.05 wt% I_2_.^[^ ^2h^ ^]^ The battery achieved a remarkable capacity of 1105 mAh g^−1^ and a peak energy density of 502 Wh kg^−1^ at 100 mA g^−1^ (**Figure** **7**a). The inclusion of the I_2_ additive significantly boosts both the reaction kinetics and the overpotential for the cycling of the cathode and Zn.

2) *Ethylene glycol*: Guo et al. introduced the novel approach by incorporating ZnI_2_ as an additive and employing ethylene glycol (EG) and Zn(CF_3_SO_3_)_2_ as co‐solvent electrolyte, targeting the challenges related to zinc dendrite growth and sulfur byproduct formation (SO_4_^2−^ and HS^−^), thus prolonging battery lifespan.^[^ ^45^ ^]^ The presence of EG was found to introduce stronger hydrogen bonds, effectively restraining sulfur side reactions and enlarging the electrolyte's electrochemical window. Furthermore, the interaction of EG with Zn ions enhances ionic conductivity within the solution. The battery configuration utilized nanoporous carbon as the cathode (S@NPC) (Figure 7b), and demonstrated superior performance, delivering an excellent capacity of 1435 mAh g^−1^ at 0.1 A g^−1^ and maintaining a capacity above 300 mAh g^−1^ following 250 cycles, which underscored the efficacy of this electrolyte optimization strategy for improving AZSBs longevity and efficiency.

3) *Tetraglyme*: In the study conducted by Yang et al., Zn(OTF)_2_ and tetraglyme (G4) are utilized as co‐solvents with I_2_ (additive) introduced to the electrolyte.^[^ ^31^ ^]^ This combination notably yields a synergistic effect that enhances battery performance. Specifically, the presence of G4 molecules acts as a barrier to water migration into the cathode, which facilitates the mobility of zinc ions and curtails the generation of SO_4_^2−^. Moreover, the collaborative interaction between G4 and I_2_ significantly boosts the solid‐solid transition of sulfur within the cathode (Figure 7c). This synergy catalyses a higher efficiency in the solid‐solid conversion of sulfur and contributes to an impressive increase in the reversible capacity of batteries (775 mAh g^−1^ at 2 A g^−1^).

4) *PEG‐400*: One of the challenges in sulfur conversion during charging is the uneven accumulation of ZnS, which forms during the discharge phase. Zhou et al. addressed this issue by incorporating PEG‐400 into a 1 M Zn(Ac)_2_ solution.^[^ ^78^ ^]^ Adding PEG (when the ratio of mass between H_2_O and PEG‐400 is 2:5) facilitates the solvation of Zn^2+^ ions will create a distinctive solvation structure that encourages the formation of uniform, finely dispersed ZnS particles, averaging 180 nm in diameter (Figure 7d). Such uniformity in particle size significantly diminishes voltage hysteresis during the charge and discharge cycles, enhancing the transition efficiency of ZnS back to sulfur and, consequently, the reversibility of the reaction of cathode. The S@CNTs cathode utilized in conjunction with this aqueous electrolyte, demonstrates a great initial capacity of 1116 mAh g^−1^ (at 0.1 A g^−1^). Additionally, PEG‐400 contributes to stabilizing the anode and mitigate the dissolution of sulfur at the cathode, benefiting both electrodes simultaneously. The employment of PEG‐400 as an additive thereby endows the battery with attributes such as low overpotential and consistent reversibility, highlighting its advantageous impact on both the anode and cathode performances.

5) *Thiourea*: The study by Chang et al. highlighted the inhibited formation of SO_4_^2−^ also via the addition of thiourea (TU).^[^ ^79^ ^]^ This compound not only reduces the formation of SO_4_^2−^ but is also involved in redox reactions throughout the electrochemical cycling (Figure 7e). Specifically, the interaction between the thiourea intermediates and effectively reduces the strength of the Zn‐S bonds in ZnS within the discharge products due to its distinct positive and negative centers, thereby expediting reaction kinetics. Importantly, the intermediates of TU exhibit a markedly higher reactivity toward ZnS compared to hydrogen atoms, which plays a crucial role in suppressing SO_4_^2−^ formation. Consequently, this modification leads to the S@KB cathode demonstrating outstanding durability, evidenced by keeping 67% capacity over 300 cycles, emphasizing its promise for efficient energy storage solutions.

As highlighted earlier, highly concentrated electrolytes, deep eutectic solvents (DESs) and hybrid aqueous systems can all contribute to the enhance performance of AZSBs, one of the most important advantages is to avoid the low decomposition voltage of water in AZSBs. Increasing the concentration of solute in water will form a “water‐in‐salt” scenario to diminish the immediate involvement of water molecules in the electrochemical processes. Also, regulating electrolytes is another essential technique to alter the kinetics and thermodynamics of HER. Elevating the electrolyte concentration alters the coordination of Zn^2+^ ions and reforms the solvation sheath, which lowers the equilibrium potential of H_2_O/H_2_ and consequently inhibits HER.^[^ ^80^ ^]^ DESs can achieve an expansive electrochemical window and minimal HER by combining hydrogen bond donors and acceptors, resulting in a mixture whose melting point is lower than its constituents. Cai et al. formulated a DESs using cost‐effective urea and choline chloride (ChCl), demonstrating that this DESs can extend the electrochemical window, thus bypassing the low decomposition voltage of water.^[^ ^18c^ ^]^

Apart from this, Zhang et al. used concentrated ZnCl_2_ (30 M) to form [Zn(OH_2_)Cl_4_]^2−^ and [ZnCl_4_]^2−^ by promoting the coordination between Zn^2+^ ions and H_2_O, which pushed the onset potential of HER to a lower potential, thereby weakening HER.^[^ ^81^ ^]^ On the other hand, Wan et al. utilized a “salt‐in‐water” mixed electrolyte comprising 1 M Zn(CF_3_SO_3_)_2_ and 21 M LiN(CF_3_SO_2_)_2_ to limit the activity of water through the strong Coulombic interaction between the oxygen atoms and Li^+^ ions.^[^ ^82^ ^]^ Nonetheless, high concentrations of salt may cause side reactions, such as salting‐out effects.

In summary, it has been proven that the introduction of additives and redox mediators into the electrolyte substantially bolsters the performance of AZSBs. For selecting electrolyte additives, **Table** **2** provides detailed descriptions of different electrolyte additives. This enhancement of the electrolyte formulation directly reduces issues during charging and discharging such as sulfur side reactions, zinc dendritic, and irregular deposition products. Concurrently, the adjustments lead to marked improvements in reversible specific capacity, overall stability, and reaction kinetics. Nonetheless, the realm of solid electrolyte interface (SEI) is relatively uncharted, underscoring the necessity for further comprehensive investigation.

**Table 2:** Summary table of the electrochemical performance of the different AZSBs.

Cathodes	Electrolyte	Additives	Energy densities [Wh kg^−1^]	Initial discharge capacity [mAh g^−1^]	Cycling performance [retention/cycles/A g^−1^]	Refs.
S@KB	ZnCl_2_	–	1083.3	1668	Primary battery	[53]
S@KB	ZnSO_4_	TU	803.3/0.1 A g^−1^	1475.5/5 A g^−1^	67%/300/5	[79]
SeS_2_/PCS	ZnSO_4_	I_2_	772	1107/0.1 A g^−1^	85%/1000/5	[69]
S@FeNC/NC/CC	ZnSO_4_	–	–	1143/0.2 A g^−1^	57.7%/300/0.5	[62]
ZnS@CF	ZnSO_4_	TUI	274_ZnS_ (832_s_)/0.1 A g^−1^	197_ZnS_/5 A g^−1^	66%/300/2	[18d]
Ag_2_S	ZnSO_4_	CuSO_4_	–	510	60%/800/0.02	[67]
S@Fe‐PANi	ZnSO_4_	PVA	720/0.2 A g^−1^	1205/0.2 A g^−1^	85%/500/2	[16a]
S@C	ZnSO_4_	Gelatin/CuSO_4_	2372	3084	78%/100/0.5	[76]
S@CNTs‐50	Zn(Ac)_2_	I_2_	502/0.1 A g^−1^	1105	85%/50/1	[2h]
S@CNTs‐50	Zn(Ac)_2_	PEG‐400	–	1117/0.1 A g^−1^	82%/300/1	[68]
CNF‐S	Zn(Ac)_2_	EG/I_2_	–	667 /0.5 A g^−1^	95%/2000/5	[78]
S/Zn–NHPC	Zn(Ac)_2_	CuSO_4_/Na_2_SO_4_	1995/1 A g^−1^	2250/1 A g^−1^	94.5%/500/1	[63]
S@NPC	Zn(OTF)_2_	EG/ZnCl_2_	730/0.1 A g^−1^	1435	70%/250/ 3	[45]
CMK‐3@S	Zn(OTF)_2_	–	–	788/0.2 A g^−1^	90%/200/1	[54]
HCS/S‐53.7	Zn(OTF)_2_	I_2_/G4	–	775/2 A g^−1^	70%/600/4	[31]
PAC/S‐60.33%	Zn(OTF)_2_	I_2_/G4	297.5	633.5/0.5 A g^−1^	40%/400/5	[18b]
HMCs‐3@S	Zn(OTF)_2_	–	–	591/0.1 A g^−1^	98%/100/1	[57]
LF‐PLSD	Zn(TFSI)_2_	–	274_ZnS_ (832_s_)/0.1 A g^−1^	1148/0.3 A g^−1^	54%/700/1	[12b]
CoS@SS	Na_2_S_4_	NaOH	586	966	85%/50/0.005	[46]
NiS/Cu_2_S/KB	KOH + ZnO	CuSO_4_	722.4/1 A g^−1^	1073/1 A g^−1^	89.6%/250/1	[66]

## 5 Applications of AZSBs

In spite of the rapid advancements and widespread application in transportation, portable electronics, electric cars, and intelligent grid systems, LIBs face significant challenges due to their instability and flammability, leading to safety concerns and numerous fire incidents.^[^ ^83^ ^]^ At the same time, the rising demand for flexible electronics has brought to light the limitations of current LIBs, which predominantly feature prismatic or cylindrical shapes encased in rigid structures, thereby rendering them less suitable for integration into portable and wearable technologies.^[^ ^84^ ^]^ This situation creates a crucial opportunity for the investigation and advancement of zinc‐ion batteries ZIBs. ZIBs offer distinct advantages over LIBs by being more cost‐effective, more abundant reserves, and less toxic. Thus, ZIBs emerge as a promising candidate to supersede LIBs in the future energy storage landscape, heralding a new era of battery technology.^[^ ^85^ ^]^

**Figure 8:**
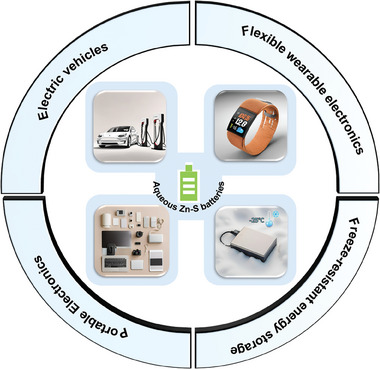
The schematic diagram of various applications of the aqueous Zn‐S batteries.

For example, solid‐state Zn‐Mn batteries utilizing a chosen polymer electrolyte(PAM) have been leveraged to extend secure wearable devices.^[^ ^86^ ^]^ The specific electrolyte develops the mechanical robustness and ionic conductivity of the battery and develops applications in stretchable yarn zinc‐ion batteries (ZIBs) to offer greater flexibility.^[^ ^87^ ^]^ Such an approach positions the Zn‐Mn batteries as an alternative to explosive and expensive LIBs.^[^ ^88^ ^]^ Therefore, it is widely used in extensive applications in wearable and implantable equipment, fitness trackers, and smartwatches.^[^ ^19^, ^89^ ^]^ However, the actual capacities observed in intercalation‐based cathodes of ZIBs (such as Prussian blue and MnO_2_, etc.) generally fall within a modest range of 50 to 300 mAh g^−1^.^[^ ^90^ ^]^ This discrepancy significantly constrains the energy density achievable by ZIBs. The prospective use of sulfur as the cathode material with its ultra‐high theoretical capacity of 1675 mAh g^−1^ casts a promising spotlight on the future of the development of ZIBs. Furthermore, prioritizing safety and environmental considerations, the aqueous electrolytes employed in the batteries are formulated from biocompatible chemicals with minimal toxicity. Additionally, the performance of electrolytes can be further optimized by mixing water with other solvents or additives to be deployable in extreme environments (under 0 °C). There are some applications of AZSBs (**Figure** **8**):

1) *Flexible Wearable Electronics*: the outstanding capacity retention of AZSBs under diverse bending scenarios enhances their appeal for flexible wearable devices. In the innovative work conducted by Sonigara et al., a flexible aqueous gel Zn‐S battery featuring a cathode composed of sulfur‐decorated Ti_3_C_2_T_x_ (S@Ti_3_C_2_T_x_) was developed.^[^ ^18e^ ^]^ This battery not only delivers impressive performance with a current density of 300 mA g^−1^ with a good specific capacity of 772.7 mAh g^−1^ but it has also been successfully series‐integrated to energize a diverse array of electronic devices, including digital clocks, LEDs, and robots. Based on the advantages mentioned above, AZSBs are well‐suited for powering wearable health monitoring devices. For example, in projects involving smartwatches that can track heart rate, and monitor sleep quality and physical activity levels, AZSBs fulfil the criteria for devices that demand to be lightweight, flexible, and capable of delivering consistent power over extended periods. AZSBs have flexible and durable electrodes, making them ideal for wearable devices that need to bend and flex in daily use, and their exceptional charge retention along with high energy density means that smartwatches can operate for weeks on a single charge avoiding the inconvenience of frequent charging. These breakthroughs laid fresh groundwork for establishing flexible energy storage systems, underscoring the significant potential of AZSBs in real‐world applications.

2) *Freeze‐Resistant Energy Storage*: Unlike the costly, toxic, and flammable electrolytes found in LIBs or LSBs, AZSBs equipped with hydrogel electrolytes offer a non‐toxic, cost‐effective, and notably anti‐freezing energy storage solution, enabling their use in low‐temperature environments. Amiri et al. successfully incorporated EG and I_2_ as redox mediators in the electrolyte of AZSBs, creating a hydrogel with antifreeze properties. This innovation ensured the stability and functionality of AZSBs within a wide temperature range from −25 to +25 °C.^[^ ^91^ ^]^ Incorporating EG into the hydrogel electrolyte notably lowers the freezing point, allowing operation in temperatures down to −25 °C to prevent the electrolyte from solidifying in cold environments. The action of EG not only safeguards against the crystallization of water within the electrolyte but also preserves its electrochemical stability and augments its compatibility with the hydrogel's polymer framework. By adding I₂ as a redox mediator, the process of stripping/plating is stabilized, and charge polarization is diminished from 0.4 to 0.2 V, effectively lowering the activation energy required, enhancing the kinetics of the electrochemical reactions, and discernibly boosting the capacity. Ensure energy storage devices can be used under special conditions. Such as at Antarctic research stations in low‐temperature environments, traditional batteries often experience performance degradation and a heightened risk of failure. However, AZSBs have demonstrated the ability to retain operational efficiency even at temperatures down to −25 °C, it can be used to power sensors in cold conditions. For sensors used in glacier monitoring projects to collect data on temperature, humidity and ice movement, AZSBs can still provide reliable power to the sensors without frequent replacement or maintenance and showcase the durability and dependability of AZSBs in facing the rigors of extreme environmental conditions.

3) *Electric Vehicles*: The rise of electric vehicles has significantly alleviated the issue of fossil fuel scarcity, but the adoption is hampered by the battery life of electric vehicles is far inferior to that of internal combustion engine vehicles. The transition to AZSBs from the current lithium batteries promises to enhance safety, lower costs, and, thanks to their superior theoretical capacity, extend the driving range of EVs. Should these developments prove successful, AZSBs have the potential to become a leading power source for the future of electric vehicle technology.

4) *Portable Electronics*: AZSBs with superior electrochemical characteristics including a high theoretical capacity and rapid charging capabilities, stand out as a prime option for fueling the upcoming wave of portable electronic devices. Their eco‐friendly nature and enhanced safety traits significantly reduce the likelihood of combustion during storage, positioning them as a safer energy solution.

The many advantages reflected in the application of AZSBs will also become a promising development area in the energy market. The positioning in the current energy market can be based on the following points:

1) *Safety and Environmental Considerations*: Aqueous zinc‐sulfur batteries use water‐based electrolytes, which inherently make them safer than LIBs and less prone to thermal runaway and explosion events. The eco‐friendly nature of zinc and sulfur further streamlines battery recycling processes.

2) *Cost‐Effectiveness*: Unlike the need for cobalt and nickel in most LIBs technologies, the materials used in AZSBs are mostly cheap, and this economic advantage is one of the important considerations for large‐scale energy storage applications.

3) *Durability and Flexibility*: AZSBs show good cycle life and stability even at low temperatures. From small consumer products to large grid‐scale energy storage systems, their scalability can further increase their position in the energy market.

In addition, AZSBs are better able to meet the needs of specific industries. Integrating renewable energy sources such as solar and wind power into the grid requires reliable and cost‐effective energy storage solutions. The safety, affordability and scalability of AZSBs make them very suitable for off‐grid energy storage systems. Especially in remote areas, its stability and low maintenance have significant advantages over traditional lead‐acid batteries. Furthermore, In the realm of electric vehicles, where long‐range capabilities are crucial, AZSBs present an advantageous alternative to LIBs, courtesy of their stability and high energy capacity, making a viable choice as the sector evolves toward prioritizing safety and efficiency in charging.

Although current challenges limit AZSBs utilization, the high theoretical capacity, and other benefits remain undeniable. Progress in AZSBs technology development promises broader deployment to satisfy the energy demands of diverse devices in the future.

## 6 Summary and Outlook

In summary, owing to the benefits of Zn including its plentiful reserves and affordability, high theoretical capacity, and low redox potential, AZIBs are the most likely to replace LIBs in the future. Selecting sulfur as a cathode boosts the capacity and energy density of ZIBs and enhances environmental sustainability. Research on AZSBs is primarily focused on two major areas: electrolyte modification and cathode optimization, both crucial for enhancing battery performance. The discussion is structured first to classify the strategies of cathode optimization, which are categorized into targeting either the modification of host carbon materials or the development of hybrid cathodes. Then it is followed by describing in detail the choice of electrolyte, and the impact of various factors, including solute, concentration, and solvent, on the efficiency of AZSBs is outlined. Nevertheless, the practical deployment of such batteries faces hurdles including sluggish reaction kinetics, the inherently low conductivity of sulfur cathodes, volumetric expansion during charging and discharging cycles, corrosion, hydrogen evolution, and dendritic growth on zinc anodes. It is currently in the developing phase, with the urgent need to address challenges and blockages:

1) The mechanism of AZSBs remains partly unresolved, particularly the operational foundations of certain systems. Central to this uncertainty is the transition between S and ZnS whether it involves the direct conversion of elemental sulfur to S^2−^ or the formation of an intermediary polysulfide chain. To achieve a more comprehensive understanding, future studies should integrate theoretical models with experimental data such as XAS results.

2) During the conversion process, the sulfur cathode faces the problems of generating insulating products (ZnS), consuming sulfur inside reactions to generate irreversible products (SO_4_^2−^), and volume expansion, resulting in slow reaction kinetics and shortened cycle life. These challenges can be overcome by referring to the original design of sulfur cathode for lithium‐sulfur batteries, such as incorporating nitriding or carburizing procedure for molybdenum nitride or carbide carbonized encourage the ingress of electrolytes and facilitate the movement of ions to improve cathode performance.^[^ ^92^ ^]^ This method can also be applied to sulfur in AZSBs to boost redox kinetics. Furthermore, using TMDs (transition metal dichalcogenides) instead of carbon as the host materials can display better electrical conductivity properties and 2D‐layered structure TMDs can limit the sulfur volume expansion during charge‐discharge cycles.^[^ ^93^ ^]^

3) While enhancing battery performance is possible by adding appropriate additives into the aqueous electrolyte, irreversible side reactions remain unavoidable due to the intrinsic nature of the aqueous solution. A shift toward a non‐organic electrolyte that eschews water can eliminate HER and the battery life will be extended. Using gel electrolytes as alternatives to aqueous solutions addresses both the issue of water evaporation within the electrolyte and prevents electrolyte leakage, especially in the context of flexible and wearable electronics.

4) The anode in AZSBs consistently encounters the same issues as the anode in ZIBs, including dendrite formation, HER, and corrosion, which severely affect the longevity of the battery. Addressing these problems is possible through the creation of robust and effective solid electrolyte interfaces (SEIs). Utilizing materials that fortify the SEI and prevent the formation of zinc dendrites, can be achieved by incorporating ZnF_2_ or Zn_3_(PO_4_)_2_.^[^ ^94^ ^]^ Also, applying protective coatings, such as ZnO, can efficiently mitigate side reactions, thus enhancing the performance of AZSBs.^[^ ^95^ ^]^

5) Few currently employed strategies take both considerations effectively to improve electrochemical performance through simultaneous modification of both electrodes and electrolytes, particularly because the problems present in both the cathode and anode cannot be addressed concurrently, potentially hindering the advancement of AZSBs. It might be worthwhile to explore the development of multifunctional approaches or the integration of various tactics to address both issues at the same time.

6) The electrochemical testing conditions should better reflect real‐world applications, including harsher environments. This involves increasing the mass loading of cathode materials, raising the current density, and reducing the amount of electrolyte used. Additionally, incorporating tests using full batteries as performance evaluation methods is essential to advance the development of AZSBs.

7) Compared with traditional liquid electrolyte batteries, AZSBs composed of solid electrolytes reduce the risk of leakage and can provide better chemical and electrochemical stability, such as avoiding the dissolution of active substances. It also mitigates the formation of zinc dendrites that can penetrate separators and cause short circuits, by providing a physical barrier to dendrite growth. Nonetheless, integrating solid electrolytes into AZSBs presents challenges. Solid electrolytes typically exhibit lower ionic conductivity compared to their liquid counterparts, leading to high interfacial resistance with electrodes that impair ion exchange, reducing battery efficiency and power. Furthermore, the complexity of producing solid electrolytes poses a significant hurdle for scaling up to meet the demands of large‐scale battery production.

8) Further research is needed to optimize the formulation of hydrogel electrolytes for even wider temperature ranges, making them viable for use in both extremely high and low‐temperature environments. For example, advanced polymer networks: Enhancing the performance and flexibility of new AZSBs across a broad temperature range calls for hydrogel electrolytes with improved stability and freeze‐resistance, which depends on the polymer chains, so complex polymer synthesis is required. Wang et al. crafted a robust hydrogel electrolyte using xanthan gum blended with a ZnCl_2_ solution, enabling operation at temperatures down to −40 °C.^[^ ^96^ ^]^ The approach of coupling the electrolyte with a high‐performance ammonium vanadate cathode and a thin Zn anode simplifies synthesis and leans toward eco‐friendliness due to the straightforward addition of polymer chains. Additionally, cross‐linking strategies: Tailoring cross‐linking practices to accommodate thermal fluctuations without compromising structural integrity is essential. The implementation of reversible or dynamic cross‐links can impart the necessary elasticity for enduring wide temperature ranges. Hong et al. introduced a versatile, multi‐component cross‐linked hydrogel electrolyte, improved with polyacrylamide and dimethyl sulfoxide (DMSO), facilitating the stable operation of ZIBs from 60 to −40 °C.^[^ ^97^ ^]^ This approach broadens temperature adaptability and opens new avenues for augmenting the range of temperature.

Even if numerous bottlenecks in the research on AZSBs, outstanding studies continue to make their way to the forefront. To make AZSBs achieve commercial viability swiftly, future research needs to explore the reaction process and mechanism more deeply. And testing the performance of the battery under real‐world use. In actual application, loading mass and stability are crucial for a thorough assessment of the overall performance.

## Conflict of Interest

The authors declare no conflict of interest.
